# Study of Water Absorption in Raffia *vinifera* Fibres from Bandjoun, Cameroon

**DOI:** 10.1155/2014/912380

**Published:** 2014-01-23

**Authors:** N. R. Sikame Tagne, E. Njeugna, M. Fogue, J.-Y. Drean, A. Nzeukou, D. Fokwa

**Affiliations:** ^1^Laboratory of Industrial and Systems Engineering Environment (LISIE), IUT/FV Bandjoun, University of Dschang, Cameroon; ^2^Laboratory of Mechanics and Modeling of Physical System (L2MSP), University of Dschang, Cameroon; ^3^Laboratory of Mechanics and Adapted Materials (LAMMA), ENSET, University of Douala, Cameroon; ^4^Laboratory of Physics and Mechanics Textile (LPMT), ENSISA, University of Haute Alsace, France

## Abstract

The study is focused on the water diffusion phenomenon through the Raffia *vinifera* fibre from the stem. The knowledge on the behavior of those fibres in presence of liquid during the realization of biocomposite, is necessary. The parameters like percentage of water gain at the point of saturation, modelling of the kinetic of water absorption, and the effective diffusion coefficient were the main objectives. Along a stem of raffia, twelve zones of sampling were defined. From Fick's 2nd law of diffusion, a new model was proposed and evaluated compared to four other models at a constant temperature of 23°C. From the proposed model, the effective diffusion coefficient was deduced. The percentage of water gain was in the range of 303–662%. The proposed model fitted better to the experimental data. The estimated diffusion coefficient was evaluated during the initial phase and at the final phase. In any cross section located along the stem of Raffia *vinifera*, it was found that the effective diffusion coefficient increases from the periphery to the centre during the initial and final phases.

## 1. Introduction

The raffia is a plant which is generally found in the tropical zones and more precisely in the Amazonia, in tropical Africa and Madagascar [1, 2]. This plant belongs to the family of monocotyledon palm trees named Arecaceae. We distinguish about twenty species of raffia in the world [3] among which there is the Raffia *vinifera*. This type of raffia does not contain a trunk [4] and essentially grows in the bottom of the mountainous and swamp areas. The Raffia *vinifera* is composed of several parts, namely, a stump, a stem, sheets, and fruits [4].

We notice that the realization of art and craft products such as the baskets, stools, hats, clothing, braces, and beds requires the Raffia *vinifera* as raw material. However, the increasing demand of these products by the population is becoming very important. Thus, the raffia forests have many interesting advantages and the regeneration time of their young plant is not short. This account for the progressive disappearance of these forests implies a problem to the environment.

The realization of the biodegradable composites containing Raffia *vinifera* fibres as reinforcement can enable us to face such a situation. During the implementation of such piece of art and craft products, we observe that the craftsmen generally take fibres on the level of the raffia stem. From these remarks, we are interested in fibres resulting from the stem for the elaboration of such composite.

Many works have been carried out on the raffia, among which the use of the bamboo raffia as braces in the concrete [5] and the study of the thermal properties of the trunk of raffia hookeri used like ceiling material [6]. On the raffia textilis, reflections have been done on the microstructure and the physical properties of fibres resulting from the sheets on the drying kinetics of those fibres whose sheets are used as materials for roofing [7, 8]. The study on the long-term behaviour of the stem of Raffia *vinifera* in compression or in flexion was approached [9–12]. The determination of some mechanical properties of Raffia *vinifera* fibres resulting from the stem such as Young modulus and density was evaluated [13].

In order to improve the knowledge on the Raffia *vinifera*, we are interested in the hydration phenomenon of these fibres. Such works on fibres resulting from the stem of Raffia *vinifera* have not yet been studied. The objectives of this work are to study the phenomenon of water absorption by determining the rate of water absorption, to develop a mathematical model and evaluate the diffusion coefficient in fibres along the stem of Raffia *vinifera*.

## 2. Materials and Methods

### 2.1. Materials

The Raffia *vinifera* fibres on which our study is based come from the stems of Raffia *vinifera* of the swamp area located at the surrounding of the University Institute of Technology Fotso Victor of Bandjoun in the west region of Cameroon. The fibres obtained were made by mechanical method as described [13]. The selected stems were those whose moisture content was within the interval 12–16%.

### 2.2. Methods

**Figure 1:**
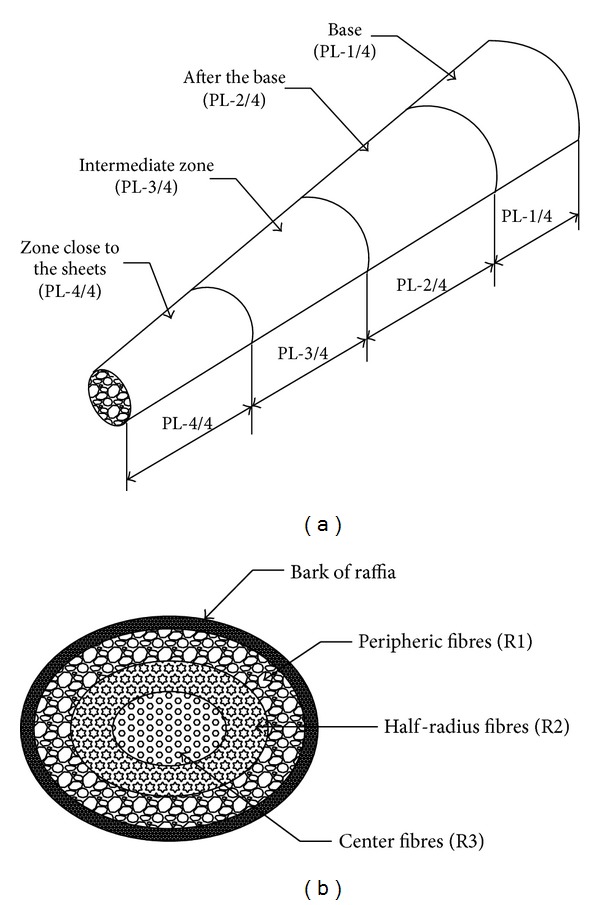
Localization of the zones of sampling of fibres along the stem of Raffia *vinifera*. (a) Longitudinal position, (b) cross section according to a precise longitudinal position.

The fibers contained in the various packages have a length of 150 mm and mass ranging from 0,50 g to 0,70 g per package. These packages of raffia fibres were taken from the twelve zones of extraction localized along the raffia stem and according to each fine cross section as shown in Figures 1(a) and 1(b). Four (4) longitudinal positions (PL-1/4, PL-2/4, PL-3/4, and PL-4/4) and three (3) radial positions (R1, R2, and R3) are shown in Figures 1(a) and 1(b), respectively.

We used a numerical balance whose precision is about 0,01 g to weigh the samples. A drying oven of *Memmert* mark was used to make the fibres anhydrous. Distilled water at the temperature of 23°C was used to immerse the various packages of fibres as it was done during the work on water absorption of some varieties of wood [14]. A dry fabric (cotton wool) was used to remove water at the surface of fibres before the next weighing after the first immersion as it was the case of [15, 16] for water absorption by food products. By the help of the software Matlab R2009b with a rate of confidence of 95%, the various experimental curves and their various models were reproduced.

To choose a mathematical model for our fibres, we carried out the tests on the various existing models and that proposed in a precise zone of the stem. Thus, the choice of the suitable model to describe this phenomenon was the one which presented the higher correlation coefficient (*R* ^2^), the lowest root means square error (RMSE) and chi-square (*χ* ^2^). These statistical parameters are defined by the following relations:

(1) $$\begin{array}{c} \text{RMSE}=\sqrt{\frac{\sum_{i=1}^{n}{\left(m_{r,i}-m_{p,i}\right)}^{2}}{N}},\\ \chi^{2}=\frac{\sum_{i=1}^{n}{\left(m_{r,i}-m_{p,i}\right)}^{2}}{N-n}, \end{array}$$

where *m*_*r*,*i*_, *m*_*p*,*i*_, *N*, and *n* are, respectively, the *i*th experimental masses, the *i*th theoretical masses, the number of observations and the number of constants.

Each package of fibres was introduced into the drying oven regulated at a temperature of 105°C ± 5°C until the mass of each package was constant. The objective is to eliminate natural water in the fibres before the study of water absorption of fibres [15–18].

After removing the water on the surface of the different fibres, we immersed samples in distilled water at constant temperature of 23°C. During regular time intervals, we measure the different weights of the sample until we reached the mass of saturation for which the mass of the package of fibres remains constant. This principle is used for the hydration of the grains of amaranth [19] or for the rehydration of the dry products [20]. In order to weigh, we remove the package of fibres from water and drop it on the dry fabric to absorb surface water. Then, we weigh each sample and reintroduce it in distilled water immediately. We start again the same process several times until obtaining a constant mass which indicates that the fibre is saturated. During the work on the hydration or the rehydration of the vegetable products and composites, this style was adopted [15, 17, 18, 21–23].

From the different weights and reaching at the saturation mass for each package of Raffia *vinifera* fibres, we can determine the water absorbed or water absorption ratio according to time.

In each zone of study, tests are done on two samples. The final mass is obtained when the mass of fibres becomes constant. The duration of immersion is estimated about 600 hrs which correspond to 25 days.

#### 2.2.1. Theory on the Diffusion of Mass through a Solid

The equation of mass transfer through a material results from the second Fick's law, which is given by

(2) $$\begin{array}{c} \frac{\partial C}{\partial t}=\mathrm{div}\left(-D\vec{\text{grad}C}\right), \end{array}$$

where *C* (mol·m^−3^) is the concentration in diffusing molecule and *D* (m^2^·s^−1^) is the diffusion coefficient.

To simplify, the fibres are considered as cylinder, in spite of the fact that they have elliptic section [13].

Equation (2) can only be written in cylindrical coordinates. We have

(3) $$\begin{array}{c} \frac{\partial C}{\partial t}=\frac{1}{r}\left\{\frac{\partial }{\partial r}\left(rD\frac{\partial C}{\partial r}\right)+\frac{\partial }{\partial \theta }\left(\frac{D}{r}\frac{\partial C}{\partial \theta }\right)+\frac{\partial }{\partial z}\left(rD\frac{\partial C}{\partial z}\right)\right\}. \end{array}$$

By taking into consideration the ratio of the length with the diameter of a fibre, we suppose that it is an infinite cylinder as it was the case for some plants' fibres [24]. Thus, (3) is reduced to

(4) $$\begin{array}{c} \frac{\partial C}{\partial t}=\frac{1}{r}\left\{\frac{\partial }{\partial r}\left(rD\frac{\partial C}{\partial r}\right)\right\}. \end{array}$$

By considering the boundary conditions, we have

 For *t* = 0, *C* = *C*_1_, 0 < *r* < *r*_*e*_.
 For *t* > 0, *C* = *C*_0_ at *r* = *r*_*e*_.

The solution for (4) can be written according to [25] as follows:

(5) $$\begin{array}{c} \frac{C-C_{1}}{C_{0}-C_{1}}=1-\frac{2}{r_{e}}\overset{\infty }{\underset{n=1}{\sum }}\frac{\exp\left(-D\alpha_{n}^{2}t\right)J_{0}\left(r\alpha_{n}\right)}{\alpha_{n}J_{1}\left(r_{e}\alpha_{n}\right)}, \end{array}$$

with *J*_0_ and *J*_1_ being, respectively, Bessel functions of zero and first order.

Let *M*_*t*_ and *M*_*∞*_ be the quantities of water diffused through raffia fibre, respectively, at the moment *t* and *t* = *∞*. Equation (5) can be rewritten for the case of the water gain rate received in terms of effective diffusion coefficient (*D*_eff_).

According to [25], (5) becomes

(6) $$\begin{array}{c} \frac{M_{t}}{M_{\infty }}=1-\overset{\infty }{\underset{n=1}{\sum }}\frac{4}{r_{e}^{2}\alpha_{n}^{2}}\exp\left(-D_{\text{eff}}\;\alpha_{n}^{2}t\right), \end{array}$$

with (*aα*_*n*_) being the positive roots of the Bessel function of zero order, a the radius of fibres, and *D*_eff_ the effective diffusion coefficient. Equation (6) was used during the mass transfer [26, 27].

#### 2.2.2. Percentage of Water Absorbed

The calculation of the percentage of water absorbed (WA) is given by the following relation:

(7) $$\begin{array}{c} \text{WA}=\frac{\left(m_{f}-m_{i}\right)}{m_{i}}\times 100, \end{array}$$

where *m*_*f*_ and *m*_*i*_ are, respectively, the final mass and initial anhydrous mass of fibres.

#### 2.2.3. Study of the Kinetics of Water Absorption of Fiber

The water absorption ratio known as *g*(*t*) is defined by

(8) $$\begin{array}{c} g\left(t\right)=\frac{M_{t}}{M_{\infty }}=\frac{m_{t}-m_{0}}{m_{\infty }-m_{0}}. \end{array}$$

Combining (7) and (8) gives

(9) $$\begin{array}{c} g\left(t\right)=\frac{M_{t}}{M_{\infty }}=\frac{m_{t}-m_{0}}{m_{\infty }-m_{0}}=1-\overset{\infty }{\underset{n=1}{\sum }}\frac{4}{r_{e}^{2}\alpha_{n}^{2}}\exp\left(-D_{\text{eff}}\;\alpha_{n}^{2}t\right), \end{array}$$

where *m*_0_, *m*_*t*_, and *m*_*∞*_ are the mass at, respectively, the initial time, *t* the actual time, and *t* = *∞* the long term. Equation (9) was used during the study of water absorption of hybrid [28] or dental composites [29].

(*1) Determination of the Effective Coefficient of Diffusion*. In this section, we will suppose that the fibres have a cylindrical form with a radius *r*, and the equivalent radius *r*_*e*_ will be given by [30].

We have

(10) $$\begin{array}{c} r_{e}^{2}=\frac{A}{\pi }, \end{array}$$

where *A* and *r*_*e*_ are, respectively, the area of the cross section and the equivalent radius of raffia fibre.

*(a) Method of Fourier Number of Diffusion*. The Fourier number of diffusion (*F*_0_) is defined by the following relation:

(11) $$\begin{array}{c} F_{0}=\frac{D_{\text{eff}}*t}{r_{e}^{2}}. \end{array}$$

Let us note by *β*_*n*_ = *r*_*e*_ *α*_*n*_ the roots of the Bessel function zero order and their different values would be taken in [31].

By substituting the expression of *F*_0_ defined by relation (11) and *β*_*n*_ in (9), we have

(12) $$\begin{array}{c} \frac{M_{t}}{M_{\infty }}=1-\overset{\infty }{\underset{n=1}{\sum }}\frac{4}{\beta_{n}^{2}}\exp\left[-F_{0}\beta_{n}^{2}\right]. \end{array}$$

By knowing water-absorbed *M*_*t*_ and *M*_*∞*_ at the time *t* and at a long time, we can obtain the various corresponding Fourier numbers of diffusion of each sample through (12). We plot the variation of the various Fourier numbers with the immersion time. The slope of the straight line enables us to deduce the diffusion coefficient of the material.

*(b) Method of Fickian Diffusion*. It is the traditional model used to predict the diffusion process through a material. In (9), we replace *β*_*n*_ and obtain

(13) $$\begin{array}{c} \frac{M_{t}}{M_{\infty }}=1-\overset{\infty }{\underset{n=1}{\sum }}\frac{4}{\beta_{n}^{2}}\exp\left(-\frac{D_{\text{eff}}}{r_{e}^{2}}\beta_{n}^{2}*t\right). \end{array}$$

The experimental values of the various water gain *M*_*t*_ obtained according to time *t* permit by the use of the software Matlab R2009b the determination of the diffusion coefficient *D*_eff_  and water gain *M*_*∞*_ after the saturation point [24, 32].

*(c) Method of Dual Stage Diffusion*. This method takes into consideration the various phases observed during the study of the phenomenon of diffusion of water through a material. Each phase was characterized by a diffusion coefficient and a water gain at the saturation point. Equation (14) was deduced from relation (13) and allows us to observe the phenomenon during the initial phase and during the final phase:

(14) Mt=M1∞[1−∑n=1∞4βn2exp⁡⁡(−D1effre2βn2∗t)] +M2∞[1−∑n=1∞4βn2exp⁡⁡(−D2effre2βn2∗t)].

The different parameters in (14) are obtained by the software Matlab R2009 and the using of the different experimental data such as water gain *M*_*t*_ according to time. *D*_1eff_  and *D*_2eff_ represent, respectively, the effective diffusion coefficients at the initial and final phases. *M*_1*∞*_ and *M*_2*∞*_ are the water gain at the saturation point corresponding, respectively, to the initial and final phases [24, 32, 33]. The water absorbed at the end of the process is given by

(15) $$\begin{array}{c} M_{\infty }=M_{1\infty }+M_{2\infty }. \end{array}$$

(*2) Proposed Model*. When the time of immersion is too long, we consider only the smaller terms of the series [34]. This assumption allows considering two terms of the expression given by (9). It permits us to propose the following relation (16) as model for water gain:

(16) $$\begin{array}{c} g\left(t\right)=c-a\exp\left(-kt\right)-b\exp\left(-mt\right), \end{array}$$

where *a*, *b*, and *c* were constants and then *k* and *m* are the parameters of the water diffusion phenomenon.

Equation (16) must respect the following conditions:

(17) $$\begin{array}{c} \text{at}\;t=0,\;g\left(t\right)=0,\\ \text{at}\;t=\infty ,\;g\left(t\right)=1. \end{array}$$

The synthesis of the models to be explored in this study is given in Table 1. We will find the correlation coefficient (*R* ^2^), the relative error, and the Chi-square of the respective models and will bring out the method which satisfies the stated conditions of (17).

## 3. Results and Discussions

### 3.1. Determination of Water Absorbed

**Figure 2:**
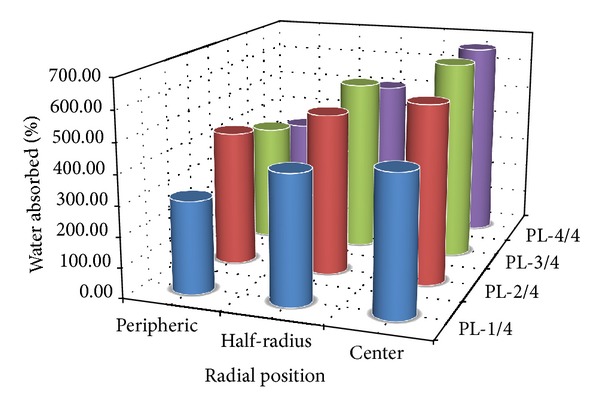
Summary of the percentage of water absorbed of Raffia *vinifera* fibres along the stem.

After evaluating the percentage of water gain of fibres in the different zones using (7), the summary of the results for the various samples is illustrated by Figure 2.

We note that the percentage of water absorption raffia fibres varies from 303% to 662% during the period of immersion estimated at 25 days. In addition, in any cross section along the stem of Raffia *vinifera*, the percentage of water gain of fibres grows from periphery towards the center.

By carrying out a comparative analysis of the different percentage of water gain shown in Table 2, it arises that one of the raffia fibres can be approximately 8 times higher than that of betel nut fibres of whose value is the smallest. Such percentage of raffia fibres will probably be due to their microstructure and could be the highest of the vegetable fibres.

### 3.2. Kinetics of Water Absorption

**Figure 3:**
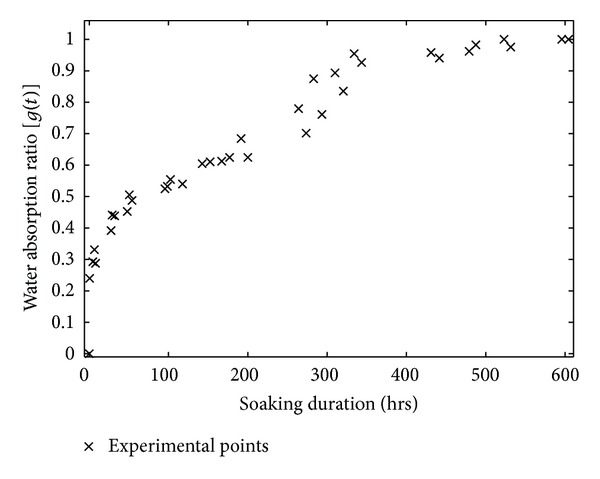
Curve of the kinetics of water absorption of Raffia *vinifera* fibres located at the half radius after the base of the stem (PL-2/4-R2).

Figure 3 presents the curves of the water gain ratio during absorption *g*(*t*), according to the time of Raffia *vinifera* fibres taken at the half radius of a cross section located at the base (PL-1/4-R2) and after it (PL-2/4-R2) along the stem.

We noticed that the curves obtained in the various zones (twelve) of the stem have the same shape as that described at Figure 3.

When observing in the curve represented in Figure 3, we notice that, during the first ten hours of immersion, the fibres reach approximately 40% of their saturation mass. The work presented the varieties of wood or plants fibres for which curves of water absorption also show a fast water gain [14, 18]. This curve also shows in the zone between 150 hrs and 300 hrs an apparent stability in water gain. This phenomenon of pseudosaturation in weight saving was also observed [15, 28, 29, 38, 42, 43].

In general, we noticed that the global form of the curve of Figure 3 is very close to the one presented [18, 22, 44] on the water gain of the composites and fibres of the plants. We have located two phases, that is, an initial phase and a final stage corresponding, respectively, to the beginning of absorption and the reach of the zone of saturation in mass. It was the consequence of the presence of two effective diffusion coefficients during the study of the hydration of the food pastes [16].

Contrary to the work on the modelling of water absorption of the date pits [39] of which the intermediate duration of saturation in water mass is approximately 300 hrs after immersion, we note on the other hand that, for Raffia *vinifera,* fibres have practically the double in terms of duration.

### 3.3. Determination of Proposed Model

**Figure 4:**
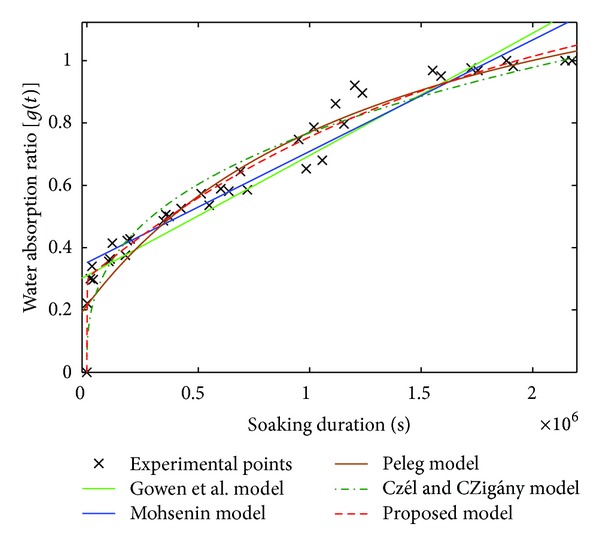
Curves of the various studied models of sample PL-1/4-R2.

Figure 4 brings out the curves of the experimental points of Raffia *vinifera* fibres of half radius coming from the base of the stem and those of the various explored models.

We notice that Gowen et al. [17] and Mohsenin [35] models did not converge when the soaking duration is too long. On the other hand, the three other models show a convergence after a long time. Similar report was made on the work of soaking of the red beans [45].

Table 3 presents the different values of the parameters obtained for each model as well as the correlation coefficient (*R* ^2^), the root means square error (RMSE), and the Chi-square (*χ* ^2^) of the samples resulting from the three zones of the cross section located at base (PL-1/4) of the stem.

By reading the values obtained in Table 3, only the proposed model gives a correlation coefficient higher than 0.97. It represents the greatest values in Table 3 compared to those of the other models. In the same way, the values of RMSE and *χ* ^2^ acquired for this case are as low as possible.

**Figure 5:**
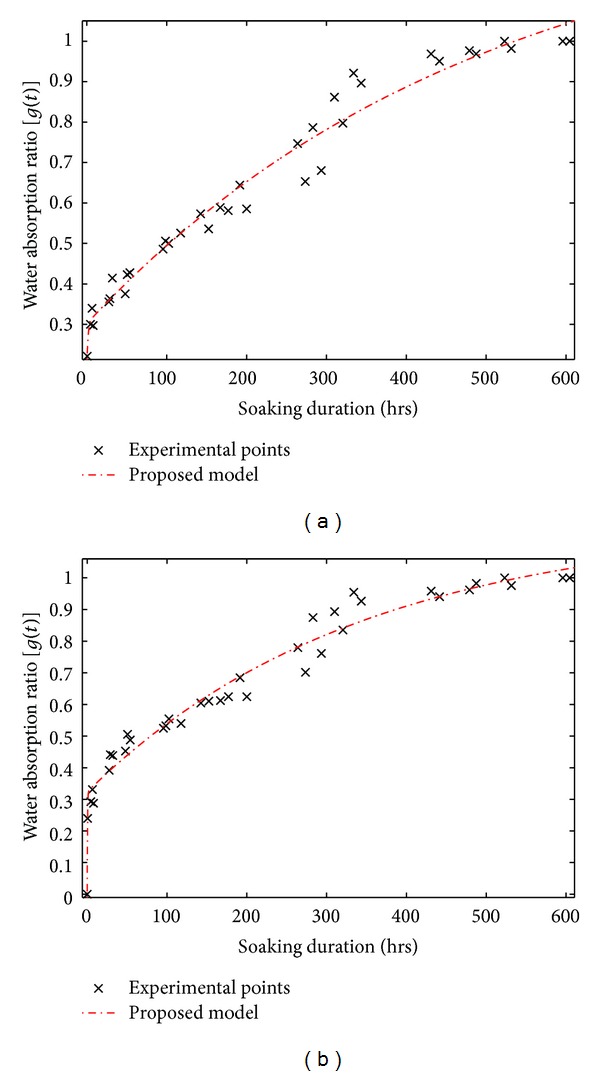
Curve of the proposed model for Raffia *vinifera* fibres located at the half radius of (a) base of the stem (PL-1/4-R2); (b) after the base of the stem (PL-2/4-R2).

The curve of the proposed model of (16), represented on Figures 5(a) and 5(b), permit us to observe that this model follows the maximum of experimental points as well as possible. This report was also made for all the other Raffia *vinifera* fibres studied in the other zones of sampling along the stem.

Table 4 illustrates the different constants of hydration (*k* and *m*) of the proposed model according to the extraction zones of raffia fibres laid down in our study (12 zones).

We notice that the values of the correlation coefficient (*R* ^2^) obtained in the different extraction zones are higher than 0.96. These values remain high compared to those of the other models on Table 3. The parameter *m*, characterizing the initial phase of absorption of water, gives values between 1.969 h^−1^ and 4.811 h^−1^, and the parameter *k* describing the final phase has values between 1.92 × 10^−3^ h^−1^ and 4.36 × 10^−3^ h^−1^.

In conclusion, we propose that the mathematical model which can as well as possible describe kinetics of water absorption of Raffia *vinifera* fibre at a constant temperature (*T* = 23°C) is defined in the following way:

(18) $$\begin{array}{c} g\left(t\right)=c-a\exp\left(-kt\right)-b\exp\left(-mt\right). \end{array}$$

### 3.4. Determination of the Effective Diffusion Coefficients

To determine the different effective diffusion coefficients, we used the Fickian diffusion method and dual-stage diffusion method.

The effective diffusion coefficient *D*_eff_, the theoretical water gain at the saturation point (*M*_*∞*_), and the coefficient of correlation (*R* ^2^) obtained for the Fickian method were gathered in Table 5 by taking into consideration the twelve zones of sampling.

We observe that, in Table 5, the coefficient of correlation (*R* ^2^) lies between 0.8314 and 0.9352.

For the method of dual-stage diffusion, the effective diffusion coefficients *D*_1eff_ and *D*_2eff_, respectively, of the initial and final phases, the theoretical water gain at the saturation point (*M*_**∞**_), and the coefficient of correlation (*R* ^2^) of each sample were inserted in Table 6.

The values of the coefficient of correlation (*R* ^2^) vary in the interval of 0.9213 and 0.9657.

**Figure 6:**
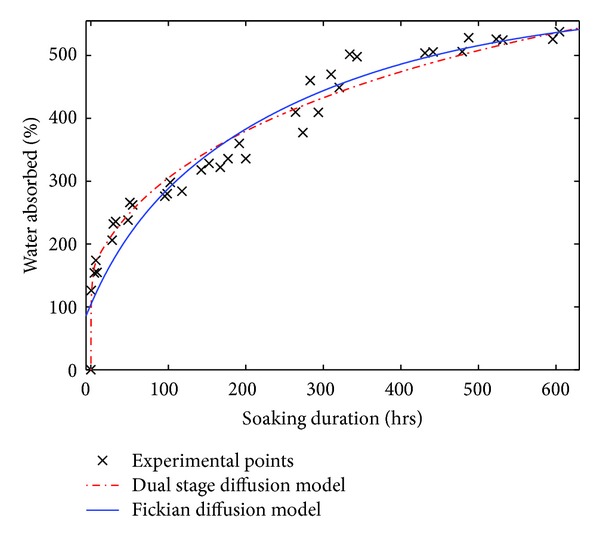
Curves of Fickian diffusion and dual stage diffusion models for the Raffia *vinifera* fibres located at the half radius after the base of the stem (PL-2/4-R2).

Figure 6 shows the graphical representation of each model of the two methods used for the choice of the way to adopt for the determination of the effective diffusion coefficient of Raffia *vinifera* fibre during the water absorption phenomenon.

We observe that the method of dual-stage diffusion model during the initial phase of water absorption fits more the experimental points compared to the method of the Fickian diffusion model.

By also analyzing the values of the coefficients of correlation (*R* ^2^) obtained in Tables 5 and 6, it comes out that the method of dual-stage diffusion gives the best results. We can say that the parameters of Raffia *vinifera* fibers during the water absorption phenomenon would be determined by the method of dual-stage diffusion model.

It can be observed from Table 6 that the effective diffusion coefficient of raffia fibres in the initial and final phases of water absorption varies, respectively, within the intervals [7.12 × 10^−5^ − 2.36 × 10^−4^] mm^2^/s and [2.87 × 10^−8^ − 6.73 × 10^−8^] mm^2^/s. This difference between the two phases can be explained by the fact that at the beginning of the water absorption, the raffia fibre absorbs water a little more quickly and eventually by the presence of the cavities inside their structure.

**Figure 7:**
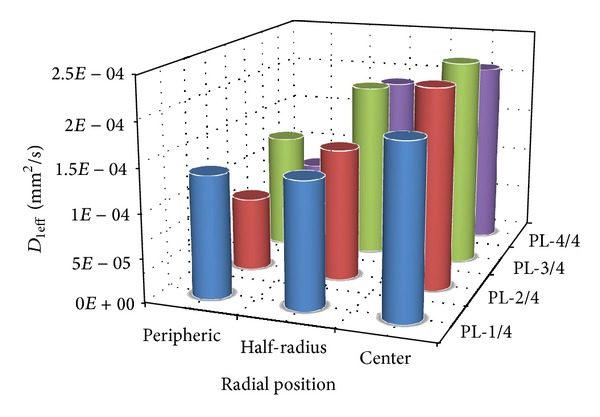
Summary of the effective diffusion coefficient of Raffia *vinifera* fibres during the initial phase of water absorption along the stem.

Figure 7 illustrates the evolution of the effective diffusion coefficient of raffia fibres in their initial phase during water absorption along the stem. We noticed that, on an unspecified cross section, the diffusion coefficient grows from the periphery towards its center. This observation can be predicted by considering the results obtained from the study of the variation of the rate of absorption of water in percentage.

**Figure 8:**
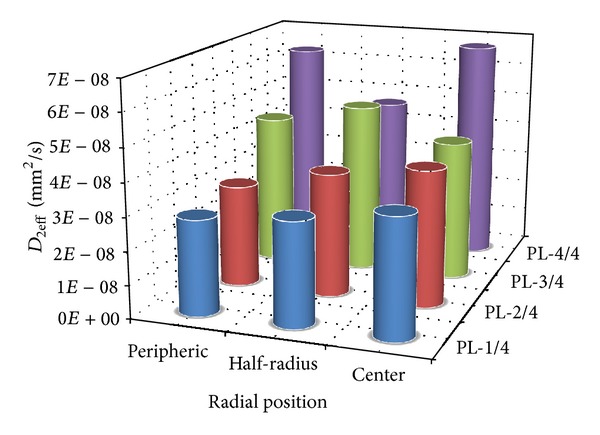
Summary of the effective diffusion coefficient of Raffia *vinifera* fibres during the intermediate or final phase of water absorption along the stem.

Figure 8 showing the effective diffusion coefficients of raffia fibres in final phase brings the same observations as previously.

By looking at the different values presented in Table 7, it is revealed that rice and corn grains and betel nut fibres have effective diffusion coefficients comparable with those obtained at the initial phase. In the same way, the effective diffusion coefficients of Raffia *vinifera* fibres were close to the values of hemp, flax, jute, and sisal fibers. On the other hand, the dried onions and the varieties of wood have values higher than those of Raffia *vinifera* fibers. This variation can be explained by the difference observed on the experimental conditions with a constant water temperature (*T* = 23°C) for Raffia *vinifera* fibers and the characteristic of their microstructure. The theoretical values of percentage of water absorption at the saturation point presented in Tables 5 and 6 are not more different to those obtained during the experimental process illustrated by Figure 2.

## 4. Conclusion 

At the end of this study which is related to the diffusion of water mass through the phenomenon of absorption by Raffia *vinifera* fibre, we evaluated the rate of water absorption (percentage) at constant temperature (*T* = 23°C) along the stem of raffia according to the extraction zones of the fibres. This rate oscillates globally between 303% and 662%. Thus, it was noted that during an immersion period in an unspecified cross section of the stem, the rate of water absorption decreases from the center towards the periphery. In addition, we established a new mathematical model which as well as possible describes the phenomenon of water absorption with a correlation coefficient (*R* ^2^) higher than 0.96. The time of immersion of the fibres before they become saturated with water is estimated approximately at 600 hrs (25 days). Then, we observed that during the water absorption, the fibres presented a pseudosaturation between 150 hrs and 300 hrs in water. All the curves presented two phases, that is, an initial phase which expresses the beginning of absorption and a final phase corresponding to the saturation in water mass by the fibres. Furthermore, we continued with the determination of the effective diffusion coefficients through the method of dual-stage diffusion Fick's law model. The first defines the speed of absorption in the initial phase whose values are between 7.12 × 10^−11^ and 2.36 × 10^−10^ m^2^/s and the other indicates the speed of absorption in the final phase whose values are also located between 2.87 × 10^−14^ and 6.73 × 10^−14^ m^2^/s. Finally, it was observed that these effective diffusion coefficients in an unspecified cross section located along the stem increase from the periphery towards the center.

## Figures and Tables

**Table 1:** Synthesis of the different models used for the study of water absorption of vegetables fibres.

Number of the model	Name of the model	Equation of the model	References
1	Mohsenin	*g*(*t*) = *a*∗[1 − exp⁡(−*b*∗*t*)] + (*c* + *d*∗*t*)	[14, 35]
2	Peleg	$g\left(t\right)=c+\frac{t}{a+b*t}$	[36]
3	Gowen et al.	*g*(*t*) = (*a* − *b*)∗exp⁡(−*k*∗*t*) + *b*	[17]
4	Czel and Czigany	*g*(*t*) = *a*∗*t* ^*m*^	[22]
5	Proposed	*g*(*t*) = *c* − *a*∗exp⁡(−*k*∗*t*) − *b*∗exp⁡(−*m*∗*t*)	Studied case

**Table 2:** Comparison of water absorbed of some natural fibres.

Types of fibers	Water absorbed (%)	Soaking duration	*T* (°C)	References
Afra wood	102			
Ojamlesh wood	54	25 days	25	[14]
Roosi wood	120			

Hemp	62			
Okra	64	13 h	(27–67)	[18]
Betel nut	38			

Raffia *vinifera *	(303–662)	25 days	23	Studied case

**Table 3:** Values of the parameters of the different models of fibres located at base (PL-1/4) of the stem of raffia.

Type of model	Radial position	*a*	*b*	*c*	*d*	*m* (h^−1^)	*k* (h^−1^)	*R* ^2^	RMSE	*χ* ^2^
Gowen et al. model	Peripheric	0.2686	1.087				3.75 × 10^−3^	0.898	0.08747	0.2678
	Half-radius	0.2234	1.161				3.17 × 10^−3^	0.9337	0.07463	0.195
	Center	0.2319	1.153				3.2 × 10^−3^	0.9223	0.08009	0.2245

Mohsenin model	Peripheric	0.3682	0.2037	7.79 × 10^−2^	1.06 × 10^−3^			0.9496	0.0624	0.1324
	Half-radius	0.3614	1.881	5.78 × 10^−5^	1.26 × 10^−3^			0.9617	0.05751	0.1125
	Center	0.3725	1.495	5.67 × 10^−4^	1.23 × 10^−3^			0.9593	0.05882	0.1476

Peleg model	Peripheric	255.5	0.9096	0.2507				0.9023	0.08561	0.2565
	Half-radius	291.7	0.7457	0.2138				0.935	0.07388	0.1911
	Center	288.9	0.7693	0.2206				0.9242	0.07913	0.2192

Czel and Czigany model	Peripheric	0.1618				0.2817		0.943	0.06447	0.1496
	Half-radius	0.108				0.3487		0.9487	0.06473	0.1508
	Center	0.1172				0.3348		0.9487	0.06419	0.1484

Proposed model	Peripheric	0.9141	0.362	1.276		3.352	2.18 × 10^−3^	0.9721	0.04709	0.07317
	Half-radius	1.054	0.3009	1.354		2.634	2.03 × 10^−3^	0.9798	0.0424	0.05932
	Center	1.06	0.3173	1.378		1.969	1.92 × 10^−3^	0.9751	0.04674	0.0721

**Table 4:** Parameters obtained for the proposed model of Raffia *vinifera * fibres along the stem.

Longitudinal position	Radial position	*a*	*b*	*c*	*m* (h^−1^)	*k* (h^−1^)	*R* ^2^	RMSE	*χ* ^2^
PL-1/4	Peripheric	0.9141	0.362	1.276	3.352	2.18 × 10^−3^	0.9721	0.04709	0.07317
	Half-radius	1.054	0.3009	1.354	2.634	2.03 × 10^−3^	0.9798	0.0424	0.05932
	Center	1.06	0.3173	1.378	1.969	1.92 × 10^−3^	0.9751	0.04674	0.0721

PL-2/4	Peripheric	0.969	0.306	1.275	2.351	2.39 × 10^−3^	0.9738	0.04888	0.07885
	Half-radius	0.8525	0.3231	1.175	2.675	2.92 × 10^−3^	0.9782	0.04357	0.06263
	Center	0.9576	0.2962	1.254	3.452	2.53 × 10^−3^	0.9758	0.04713	0.07331

PL-3/4	Peripheric	0.8275	0.3406	1.168	3.975	3.98 × 10^−3^	0.9716	0.04992	0.08225
	Half-radius	0.8266	0.3136	1.14	3.973	3.41 × 10^−3^	0.9865	0.03483	0.04003
	Center	0.9292	0.3328	1.262	2.08	2.32 × 10^−3^	0.9695	0.05116	0.08638

PL-4/4	Peripheric	0.5849	0.4593	1.044	2.996	4.36 × 10^−3^	0.9683	0.0479	0.07573
	Half-radius	0.7887	0.3504	1.139	3.423	3.30 × 10^−3^	0.9808	0.03992	0.05258
	Center	0.7922	0.3397	1.132	4.811	3.56 × 10^−3^	0.9808	0.04112	0.05581

**Table 5:** Effective diffusion coefficients and some parameters of Raffia *vinifera* fibers obtained by the method of Fickian diffusion along the stem.

Longitudinal position	Radial position	*D*_eff_ (mm^2^/s)	*M*_*∞*_ (%)	R^2^
PL-1/4	Peripheric	9.08 × 10^−8^	349	0.9013
	Half-radius	9.98 × 10^−8^	520.1	0.9309
	Center	1.06 × 10^−7^	557.2	0.9208

PL-2/4	Peripheric	8.98 × 10^−8^	528.9	0.9188
	Half-radius	1.02 × 10^−7^	586	0.9344
	Center	1.07 × 10^−7^	695.5	0.929

PL-3/4	Peripheric	1.13 × 10^−7^	432.3	0.8853
	Half-radius	1.15 × 10^−7^	625.9	0.9352
	Center	1.13 × 10^−7^	750.6	0.9148

PL-4/4	Peripheric	1.23 × 10^−7^	321	0.8314
	Half-radius	1.31 × 10^−7^	520.7	0.8981
	Center	1.34 × 10^−7^	711.8	0.9246

**Table 6:** Effective diffusion coefficients of initial and final phases and the other parameters of Raffia *vinifera* fibres obtained by the method of dual stage diffusion Fick's law model along the stem.

Longitudinal position	Radial position	*D*_1eff_ (mm^2^/s)	*D*_2eff_ (mm^2^/s)	*M*_*∞*_ (%)	*R* ^2^
PL-1/4	Peripheric	1.39 × 10^−4^	2.87 × 10^−8^	400.17	0.9574
	Half-radius	1.44 × 10^−4^	3.13 × 10^−8^	629.63	0.9577
	Center	1.94 × 10^−4^	3.57 × 10^−8^	675.03	0.9479

PL-2/4	Peripheric	8.34 × 10^−4^	3.09 × 10^−8^	626.51	0.9477
	Half-radius	1.50 × 10^−4^	3.73 × 10^−8^	672.84	0.9709
	Center	2.28 × 10^−4^	4.10 × 10^−8^	805.66	0.9566

PL-3/4	Peripheric	1.31 × 10^−4^	4.56 × 10^−8^	490.73	0.9213
	Half-radius	2.00 × 10^−4^	5.14 × 10^−8^	702.12	0.9657
	Center	2.36 × 10^−4^	4.23 × 10^−8^	872	0.9537

PL-4/4	Peripheric	7.12 × 10^−5^	4.65 × 10^−8^	367.66	0.9264
	Half-radius	1.86 × 10^−4^	4.70 × 10^−8^	598.8	0.9561
	Center	2.11 × 10^−4^	673 × 10^−8^	719.7	0.9468

**Table 7:** Comparison of the effective diffusion coefficients during water absorption.

Produced types	*D*_eff_ (m^2^/s)	References
Okra fibre	5.40 × 10^−10^	[18]
Betel nut fibre	2.80 × 10^−10^	

Pasta	5.69 × 10^−11^: initial stage	[16]
	4.20 × 10^−11^: final stage	

Hemp fibre	5.29 × 10^−12^: initial stage	[24]
	5.80 × 10^−13^: final stage	
Jute fibre	2.33 × 10^−12^: initial stage	
	2.30 × 10^−13^: final stage	
Flax fibre	2.11 × 10^−12^: initial stage	
	2.11 × 10^−13^: final stage	
Sisal fibre	4.00 × 10^−12^: initial stage	
	4.38 × 10^−13^: final stage	

Afra wood	1.38 × 10^−3^	
Ojamlesh wood	3.71 × 10^−4^	[14]
Roosi wood	4.88 × 10^−4^	

Dried onion	(1.96 × 10^−9^–8.04 × 10^−9^)	[37]

Amaranth grain	(10^−12^–10^−11^)	[38]

Date pits	9.98 × 10^−12^	[39]

Wheat grain	(1.35 × 10^−11^–6.88 × 10^−11^)	[40]

Rice grain	7 × 10^−10^	[41]

Raffia *vinifera* fibre	(7.12 × 10^−11^–2.36 × 10^−10^): initial stage	Studied case
	(2.87 × 10^−14^–6.73 × 10^−14^): final stage	
